# Therapist behaviours in a web-based mindfulness-based cognitive therapy (eMBCT) for chronic cancer-related fatigue – Analyses of e-mail correspondence

**DOI:** 10.1016/j.invent.2020.100355

**Published:** 2020-12-01

**Authors:** Anne Maas, Melanie P.J. Schellekens, Rosalie A.M. van Woezik, Marije L. van der Lee

**Affiliations:** a Helen Dowling Institute, Scientific Research Department, Bilthoven, the Netherlands; b Tilburg University, Tilburg School of Social and Behavioral Sciences, Department of Medical and Clinical Psychology, Tilburg, the Netherlands

**Keywords:** Therapist behaviours, web-based therapy, mindfulness-based cognitive therapy, Mindfulness, Cancer, Chronic cancer related fatigue

## Abstract

Web-based mindfulness-based cognitive therapy (eMBCT) has been found effective in decreasing fatigue severity in patients suffering from Chronic Cancer-Related Fatigue (CCRF). In web-based therapy, guidance from a therapist positively affects treatment outcome. So far, less is known about what kind of therapist behaviours contribute to treatment outcome. The present study aimed at 1) identifying therapist behaviours during eMBCT and 2) exploring whether these behaviours were correlated to a decrease in fatigue severity among patients. Qualitative content analyses were performed on 537 feedback e-mails from five therapists sent to 31 patients within a secured portal. Through content analyses, nine therapist behaviours were identified: emphatic utterances, probing self-reflection, informing, psychoeducation, task prompting, paraphrasing, task reinforcement, providing group context and alliance bolstering. Among these behaviours task prompting (19%), paraphrasing (16%) and task reinforcement (15%) were the most common. Linear regression analyses showed a significant association between informing and task prompting on the one hand and a decrease in fatigue severity on the other. Multivariate analysis indicated that informing and task prompting jointly explain the decrease in fatigue. These findings underline the importance for therapists to provide patients with sufficient information and to encourage them to do the exercises.

## Highlights

• We explored what therapist behaviours in eMBCT help reduce chronic cancer-related fatigue.
• Qualitative content analysis of e-mail correspondence identified nine types of therapist behaviours in eMBCT.
• Online therapist behaviours included emphatic utterances, probing self-reflection, informing, psycho-education, task prompting, paraphrasing, task reinforcement, providing group context and alliance bolstering.
• Task prompting, paraphrasing and task reinforcement were the most commonly used behaviours in eMBCT.
• Informing and task prompting were both associated with reduced fatigue six months post-treatment.

## 1 Introduction

Approximately, a quarter of cancer survivors remain severely fatigued for months or even years after completing treatment (Goedendorp et al., 2013). This persistent fatigue, termed Chronic Cancer-Related Fatigue (CCRF), can be described as “*a distressing, persistent, subjective sense of physical, emotional, and/or cognitive tiredness or exhaustion related to cancer or cancer treatment that is not proportional to recent activity and interferes with usual functioning”* (Berger et al., 2010). As CCRF hinders daily activities, it has a major impact on patients' quality of life. Therefore, effective and easily accessible interventions are needed (Bruggeman-Everts et al., 2017).

A recent meta-analysis found mindfulness-based interventions to reduce self-reported fatigue, psychological distress, depression and anxiety in cancer patients and survivors (Cillessen et al., 2019). Mindfulness can be described as intentionally paying attention to present moment experiences, in an accepting, non-judgemental way (Kabat-Zinn, 1990). Mindfulness-based cognitive therapy (MBCT) can help patients to become aware of their maladaptive feelings, thoughts and behaviours and to adjust these automatic responses to more helpful ones. As such, MBCT is ideally suited to treat CCRF as patients become more aware of their energy level during the day and potentially maladaptive coping strategies (e.g. catastrophizing about fatigue, being overactive/inactive). This awareness allows patients to choose more helpful coping strategies (e.g. accepting fatigue, balancing activities and rest) (Bruggeman-Everts et al., 2015). Due to their home-based character, web-based interventions might be more suitable for fatigued patients as they might not be able to travel due to a lack of energy or physical limitations. Therefore, Bruggeman-Everts et al. (2015) tested a web-based MBCT-program (eMBCT) for patients with CCRF in clinical practice, which proved to be effective in a randomized controlled trial (Bruggeman-Everts et al., 2017).

Similar to face-to-face therapy, the role of the therapist is of importance in web-based therapy. Meta-analyses have demonstrated the additional value of personal contact with a therapist during web-based treatments (Andersson and Cuijpers, 2009; Spek et al., 2007). For example, Spek et al. (2007) found interventions with therapist guidance to be more effective than interventions without. However, little is known about what the support of the therapist in web-based treatments entails. What we do know is that a therapeutic alliance develops in time in both MBCT and eMBCT, and that in both conditions the level of therapeutic alliance can predict reduced psychological distress and increased mental well-being post-treatment (Bisseling et al., 2019). Furthermore, Sanchez-Ortiz et al. (2011) stated that the communication from the therapist to the patient in Internet-based Cognitive Behavioural Therapy (ICBT) is mainly supportive in content rather than psychoeducational. This could be explained by the nature of web-based therapies, where psycho-education is given through online modules.

Perhaps even more interesting is the question what type of therapist behaviours relate to treatment outcome in web-based therapies. Currently, there have been two studies published on online therapist behaviours in relationship to symptom improvement. First, Paxling et al. (2013) examined 490 e-mails from three therapists interacting with 44 patients who took part in a controlled trial on ICBT for generalized anxiety disorder. By analysing the content of the written messages, eight therapist behaviours were identified. They found **task reinforcement** (i.e. behaviours aimed at reinforcing assignments already completed by the patient) to be positively associated with symptom improvement. On the other hand, deadline flexibility (i.e. behaviours related to flexibility in deadlines for submitting homework and allowing extra time to work with a particular module) was negatively associated with symptom improvement. No significant effects were found for the other identified therapist behaviours: alliance bolstering, task prompting, psychoeducation, self-disclosure, self-efficacy shaping, and empathetic utterances.

Second, Holländare et al. (2016) examined 664 e-mails from five therapists to 42 patients during the course of ICBT for depressive symptoms. They performed a qualitative content analysis resulting in nine therapist behaviours. They found three behaviours to be positively associated with symptom improvement. **Affirming** (i.e. paying attention to, acknowledging and expressing an interest in the patients' thoughts, emotions and actions, and to regard them as valid) correlated significantly with improvement in depressive symptoms at post-treatment and two-year follow-up. **Self-disclosure** (i.e. mentioning the therapists own experience and using personal examples from one's own life) and **encouraging** (i.e. therapist behaviours aimed at encouraging some type of patient behaviour) were associated with improvement of depressive symptoms directly after treatment. There were no significant associations found for the remaining identified therapist behaviours: emphasizing patient's responsibility, clarifying the framework, informing about modules, confronting, urging and guiding. Similar to Paxling et al., Holländare et al. found encouraging behaviours to be of positive influence in ICBT. However, Holländare et al. defined encouraging as both praising past behaviour and inciting future behaviour, while Päxling separated the two categories into task reinforcement (praising past behaviour) and task prompting (inciting future behaviour), hindering a one-on-one comparison. Furthermore, in contrast to Paxling et al., Holländare et al. found affirming and self-disclosure to be associated with improvement of symptoms. This might indicate that different symptoms may require different therapist behaviours.

The aim of the current study is to identify therapist behaviours during eMBCT for CCRF and to explore which therapist behaviours are associated with a reduction in fatigue. The research findings can be of importance for therapists in clinical practice, as they would be able to emphasize effective behaviours in the treatment of CCRF.

## 2 Material and methods

### 2.1 Design

In this study the therapist's written feedback on the patient’s exercises from the nine-week eMBCT-treatment was analysed. Data were gathered as part of a randomised controlled trial (Bruggeman-Everts et al., 2017) in which the effectiveness of the psychologist-guided eMBCT therapy and the physiotherapist-guided Ambulant Activity Feedback (AAF) therapy (the intervention groups) were compared to receiving psychoeducational e-mails only (the active control group). The clinical trial in which the patients participated was approved by the Twente Medical Ethical Committee (Enschede, The Netherlands), number P12–26, and was registered in The Netherlands National Trial Register under number NTR3483 (Bruggeman-Everts et al., 2017). The current study used the data of patients who completed the eMBCT treatment in a mixed design, where qualitative content analysis of online therapist behaviours was combined with a within-group design, with pre- and post-measures of patients' fatigue levels.

### 2.2 Participants

Participants were recruited via patient organizations, walk-in consultation services, social media, newspapers, and health care professionals. Participants met the following criteria: they (a) were curatively treated for cancer (all cancer types included) (b) had completed their last cancer treatment at least six months before the start of eMBCT (c) were older than 18 years; (d) scored ≥35 on the severity of fatigue subscale of the self-report Checklist Individual Strength (CIS) at baseline (Vercoulen et al., 1999); (e) had no history of psychosis or current Major Depressive Disorder and f) had followed at least six weeks of the eMBCT program. Patients who reported they had cancer recurrence or started a cancer treatment during the study were excluded from analysis (Bruggeman-Everts et al., 2015). All participants gave written informed consent prior to their inclusion in the study.

Therapists from the study site (the Helen Dowling Institute, centre for psycho-oncology) were invited to guide the eMBCT. All therapists were trained in MBCT, had experience with online therapy and worked for at least a year in the field of psycho-oncology.

The data consisted of 537 e-mail messages sent by five therapists to 31 patients. The majority of patients were women (65%) and on average they were 53.50 years old (SD = 11.00). Although all cancer types were included, the majority of patients were treated for breast cancer (41%). All patients were severely fatigued for at least three months post treatment, of which 89% had been fatigued for over a year. Four therapists (80%) were women and on average they were 52.60 years old (SD = 10.33). The mean number of e-mail messages sent from therapist to patient was 16.78 (SD = 5.59). An average message from a therapist contained 284.83 words (SD = 89.05).

### 2.3 Online mindfulness-based cognitive therapy

The intervention is a web-based psychologist-guided intervention, based on the original MBCT-program developed by Segal et al. (2002), and specifically adapted for patients suffering from CCRF. The treatment material consisted of nine eMBCT modules, which the patient could consult by logging on to their personal account. Each module involved reading weekly information about mindfulness, CCRF and/or other related symptoms (e.g. anxiety, depression and anger) and doing mindfulness exercises (body scans, sitting meditations, gentle yoga exercises and walking meditations) while listening to the MP3-files. See Table 1 for a description of each module. Furthermore, patients were encouraged to integrate MBCT in their lives by paying attention to their daily activities. They were asked to practice for six days a week and to fill out logs with their experiences with the modules. The average time they spent on the training was four hours a week.

**Table 1:** Overview of the online MBCT protocol per week.

Theme of module	Didactic teaching	Home practice
1. Automatic pilot, non-striving.	Stress-coping model, automatic pilot, coping with cancer-related fatigue	Bodyscan, eating with awareness
2. Body and breath, non-judging	Coping with pain and fatigue during bodyscan, handling thoughts during breathing exercise, tips for better sleep quality	Bodyscan with muscle tension (Jacobson), breathing exercise, awareness of activities, awareness of pleasant moments
3. Accepting boundaries, acceptance	Recognizing unpleasant experiences, awareness of handling physical and emotional boundaries, cultivating acceptance	Yoga, 3-min breathing exercise, alternated with previous exercises, awareness of unpleasant emotions
4. Patience, attention	Recognizing automatic negative cognitions, recognizing daily stress-inducing experiences and their emotional impact, promoting choices in handling daily stress.	Sitting meditation, walking with awareness, alternated with previous exercises
5. Letting go, accept things as they are	Learning how to cope with negative emotions through acceptance	Sitting practice ‘accepting what is in the present’, alternated with previous exercises, diary with negative emotions
6. Dealing with thoughts and fear, trust	Interaction of thoughts, emotions and behaviour, choosing respons, physiology of fear, fear of cancer recurrence, coping with loss	Walking with awareness, sitting meditation, alternated with previous exercises
7. Silence and compassion, loving kindness towards oneself	Planning half a day of home practice in silence, including mindfulness and compassion exercises	Additional exercises: Mountain and lake exercise, metta-mediation
8. A new perspective, taking good care of myself	Making own program of mindfulness practice without therapist feedback, list of top ten helpful cognition, accepting stress as part of life	Practice own program
9. From stress to inner strength	Repetition of previous themes	Recommended literature

Each week patients would receive a reply from the therapist within the secured portal. This reply consisted of feedback on the patient's experiences written down in the logs. The therapist supported the patient in doing the exercises and in creating a mild and open awareness for feelings, thoughts and behaviours. Each week the therapists spent approximately 35–45 min per patient. See Bruggeman-Everts et al. (2015) for more details regarding the treatment.

### 2.4 Measurement

The outcome variable, fatigue severity, was measured through the fatigue severity subscale of the self-report Checklist Individual Strength (CIS). The subscale consists of eight items, which were rated on a 7-point Likert scale. The subscale showed good reliability (cronbach's alpha = 0.88). Patients with a score of 35 on this subscale are considered to suffer from severe fatigue (Vercoulen et al., 1999).

### 2.5 Analyses

The data consisted of e-mail correspondence between therapists and their patients. First, the therapist behaviours were identified. To do so, a social scientist and behavioural scientist separately read the emails from the therapists while identifying the observed behaviour. For this procedure, we selected one complete series of e-mail correspondence of each therapist, sent to a randomly selected patient over the course of nine weeks. In this process the behavioural categories used by Paxling et al. (2013) and Holländare et al. (2016) were used as guidance to identify therapist behaviours. After coding the five documents of email correspondence independently, the two researchers compared their labels and reached consensus. The labels that best represented the data were accepted. The resulting nine therapist behaviours were then discussed and finalized with a healthcare psychologist and behavioural scientist who both work as MBCT therapists to ensure that no important behavioural aspects were missing.

The nine therapist behaviours were quantified by counting how many times each behaviour occurred on average per week. Linear regression analyses were performed to examine the association between the therapist behaviours and the decrease in fatigue severity (measured on an interval scale). For the analyses we used the different therapist behaviours as independent variables, the pre-treatment fatigue measurement as covariate and the six-months post-treatment measurement as outcome measure. Therapist behaviours that were significantly associated with the decrease in fatigue severity (p- values <0.05) were carried forward to a multivariate regression model.

The conditions of linearity, normal distribution of the residuals, homoscedasticity and the absence of multicollinearity were met (VIF < 10). However, there was a strong association between informing and task prompting (r = 0.71, p < 0.00, VIF = 1.97). The data showed that these two categories often followed each other, as the therapist regularly told the patients what to expect the upcoming week (informing) followed by an expression of encouragement (task prompting). We decided to keep the two categories separate as the VIF-score did not exceed 2.50, the two categories are theoretically different (with the aim of informing being providing information and the aim of task prompting being inciting behaviour) and as they also occurred independently of each other.

## 3 Results

### 3.1 Occurrence therapist behaviours

The therapist behaviours were divided into nine categories (see Table 2). In line with previous research we have identified emphatic utterances, psychoeducation, task prompting, task reinforcement, alliance bolstering (Paxling et al., 2013)*,* informing and paraphrasing (Holländare et al., 2016). Additionally, we have identified probing self-reflection and providing group context*.* These behaviours may be more present in MBCT, because they are key components of mindfulness-based interventions (Crane et al., 2012). Regarding probing self-reflection, MBCT is an interactive process in which the therapist takes an explorative and participatory approach to uncover habitual tendencies. Regarding providing group context, it is suggested that the therapist uses group context to underline the general nature of the mind.

**Table 2:** Therapist behaviours.

Therapist behaviours	%/M/SD per week	Description	Examples
1. Task prompting	19% M = 2.58 SD = 0.92	Behaviours prompting the patient to work with exercises and given homework. This can be done in several ways e.g.: ➢ Stimulating/motivating the patient to carry out the task; ➢ Wishing good luck; ➢ Expressing curiosity about the patients' results; ➢ Giving practical instructions how to do the task; ➢ Encouraging the patient to ask questions.	“I'm looking forward to hearing from you while working on the upcoming modules.” “Good luck with the next task.”
2. Paraphrasing	16% M = 2.29 SD = 0.76	Repeating or describing what the patient wrote down.	“You wrote that…” “I read in your message that…”
3. Task reinforcement	15% M = 2.02 SD = 0.60	Behaviours focussed on reinforcing exercises completed by the patient. This can be done in an explicit way by giving the patient a compliment or in an implicit way by acknowledging the patients' effort.	“You have described your worries clearly.” “I see that you have practiced daily.”
4. Psychoeducation	12% M = 1.69 SD = 0.53	Providing the patient with general information to better understand occurring psychological processes, the treatment goals and the purpose and meaning of the work involved in the modules.	“Thoughts will pop up in your head, whether you want it or not. That is the nature of the human mind.” “The purpose of the exercise is to notice what you experience at a particular moment, whether it is pleasant or not.”
5. Informing	11% M = 1.53 SD = 0.63	Providing the patient with practical information. This can be done by informing the patient about the organization of the module e.g. indicating when the patient can expect an answer. Informing can also take place regarding the content of the modules by referring to upcoming modules.	“I will react to your writings next Tuesday.” “Helpful thoughts will be the subject of next week's module.”
6. Empathic utterances	8% M = 1.16 SD = 0.55	Writings that show understanding and empathy for the patient's suffering, frustration or general life situation. Empathy can be given on different aspects, e.g. regarding the symptoms of the patient, training-related experiences or life events.	“That was a difficult start to your quiet day last week, with a sad message from your friend. It really does not come easy.” “I can see why respecting your boundaries is not easy for you, especially because your boundaries were different before you fell ill.”
7. Alliance bolstering	8% M = 1.06 SD = 0.49	Non-treatment specific writings that show interest in the patient's life situation and care for his or her situation. The underlying goal of this behaviour is to improve the bonding between therapist and patient.	“How were your holidays?” “Have a good weekend!”
8. Probing self-reflection	6% M = 0.80 SD = 0.45	Working in an interactive way to explore common habits and patterns and/or stimulating patients to think about themselves in different ways than usual.	“How did you deal with boundaries in the past? Did you actually feel boundaries?” “That you feel guilty about not doing the exercises and therefore do them, is perhaps also a recognizable automatic thought with an automatic response?”
9. Providing group context	5% M = 0.76 SD = 0.36	Providing the patient with the frame of reference of other people by: ➢ Giving examples of experiences of other people (e.g. experiences of other patients and/or mentioning the therapists' own experience); ➢ Describing how most people deal with a particular experience or exercise; ➢ Normalising.	“I use that exercise myself when I feel rushed.” “For a lot of patients, the sitting meditation is hard work.”

In the present study, task prompting was the most frequent behaviour. Nineteen percent of the identified therapist behaviours was dedicated to task prompting. On average therapists portrayed this behaviour 2.58 times per week. It was followed by paraphrasing (16%, M = 2.29 per week) and task reinforcement (15%, M = 2.02 per week).

### 3.2 Association between therapist behaviours and fatigue severity

The therapist behaviours informing (B = −5.95, p = 0.012) and task prompting (B = −3.53, p = 0.031) were negatively associated with fatigue severity at the end of the treatment. The therapist behaviours emphatic utterances, probing self-reflection, psychoeducation, paraphrasing, task reinforcement, providing group context and alliance bolstering were not significantly associated with fatigue severity. All associations are shown in Table 3.

**Table 3:** Results of univariate regressions with fatigue severity at 6-month follow-up as outcome.

Model	Therapist behaviour		B (SE)	T	P	R^2^
1	Emphatic utterances	Constant	30.42 (10.12)	3.01	0.006	
		Fatigue severity pre-treatment	0.21 (0.21)	0.98	0.337	
		Emphatic utterances	−3.21 (2.78)	−1.16	0.257	
		Total			0.334	0.08
2	Probing self-reflection	Constant	31.14 (9.92)	3.14	0.004	
		Fatigue severity pre-treatment	0.20 (0.21)	0.94	0.356	
		Probing self-reflection	−4.92 (3.30)	−1.49	0.148	
		Total			0.221	0.10
3	Informing	Constant	34.60 (9.25)	3.74	0.001	
		Fatigue severity pre-treatment	0.24 (0.20)	1.21	0.238	
		Informing	−5.95 (2.21)	−2.69	0.012	
		Total			0.026	0.23
4	Psychoeducation	Constant	29.15 (10.70)	2.72	0.011	
		Fatigue severity pre-treatment	0.22 (0.22)	0.99	0.333	
		Psychoeducation	−1.61 (2.90)	−0.55	0.584	
		Total			0.552	0.04
5	Task prompting	Constant	35.41 (9.82)	3.61	0.001	
		Fatigue severity pre-treatment	0.22 (0.20)	1.08	0.289	
		Task prompting	−3.53 (1.56)	−2.26	0.031	
		Total			0.061	0.18
6	Paraphrasing	Constant	29.05 (10.05)	2.89	0.007	
		Fatigue severity pre-treatment	0.25 (0.22)	1.13	0.268	
		Paraphrasing	−1.83 (2.06)	−0.89	0.382	
		Total			0.436	0.06
7	Task reinforcement	Constant	26.46 (10.33)	2.56	0.016	
		Fatigue severity pre-treatment	0.20 (0.23)	0.90	0.374	
		Task reinforcement	0.24 (2.69)	0.09	0.928	
		Total			0.640	0.03
8	Providing group context	Constant	29.99 (10.25)	2.92	0.007	
		Fatigue severity pre-treatment	0.21 (0.22)	0.95	0.350	
		Providing group context	−4.11 (4.28)	−0.96	0.345	
		Total			0.408	0.06
9	Alliance bolstering	Constant	27.96 (10.13)	2.76	0.010	
		Fatigue severity pre-treatment	0.22 (0.22)	0.99	0.332	
		Alliance bolstering	−1.50 (3.18)	−0.47	0.641	
		Total			0.575	0.04

### 3.3 Multivariate regression analysis

Including informing and task prompting in a multivariate regression analysis (see Table 4), resulted in an overall marginally significant multivariate model (p = 0.054). The individual variables informing (p = 0.149) and task prompting (p = 0.505) were no longer significant when both were added into the model.

**Table 4:** Results of multivariate regression analysis between therapist behaviours (informing and task prompting) and fatigue severity at 6-month follow-up.

Multivariate model	B (SE)	T	P	R^2^
Constant	36.20 (9.63)	3.76	0.001	
Fatigue severity pre-treatment	0.23 (0.20)	1.18	0.249	
Informing	−4.54 (3.06)	−1.48	0.149	
Task prompting	−1.41 (2.09)	−0.68	0.505	
Total			0.054	0.24

## 4 Discussion

The present study examined behaviours that therapists use during eMBCT for CCRF. Task prompting, paraphrasing and task reinforcement turned out to be the most common behaviours. This is in line with previous research (Sanchez-Ortiz et al., 2011) that found supportive behaviours to be prominent in ICBT. As the educational information is embedded in the online program, the responsibility for following the online modules and acquiring information lies primarily with the patient. As a result, the role of the therapist becomes more supportive in nature.

This study showed that informing was a positive predictor of treatment outcome. The more the therapist informed the patients, the more their fatigue reduced. Informing can be seen as a basic condition for an effective treatment, providing the patient with information about what to expect. The association between informing and outcome was not found in previous studies (Paxling et al., 2013; Holländare et al., 2016). The importance of informing in this study might come with the intense nature of eMBCT in which patients were asked to practice for at least six days a week and for four hours a week on average. To keep up with this schedule, knowing what to expect may become of greater importance.

Similar to the study by Holländare et al. (2016), we found encouraging behaviours (i.e. task prompting) to be a positive predictor of treatment outcome. The more the therapist encouraged the patients the more their fatigue diminished. However, Höllandare et al. defined encouraging as both praising past behaviour as well as inciting future behaviour. In line with Paxling et al. we made the distinction between praising past behaviour (task reinforcement) and inciting future behaviour (task prompting). Since we have separated the two categories, we were able to see that in eMBCT task prompting is more relevant than task reinforcement. Prompting patients to do homework assignments can lead them to practice more, which helps them benefit more from eMBCT. A meta-analysis (Parsons et al., 2017) across 28 studies confirmed that there was a significant association between participants' home practices in MBCT and intervention outcomes. Thus, with increased practice, better results can be achieved. Furthermore, the intensity of the eMBCT program can play a role. When practicing almost every day for nine weeks, the extent to which the therapist encourages the patient may become of greater importance**.**

These results suggest that both informing and task prompting are beneficial factors to outcome. Interestingly, when both these behaviours are added in a multivariate model the individual betas appear no longer significant. This could be due to the lack of power (small sample size) for running a multivariate regression. Moreover, informing and task prompting showed a high correlation. As such, the two independent variables may not uniquely explain the decrease in fatigue. The overlap in explained variance can, especially in case of a small sample size, result in both variables losing their significance. These results suggest that informing often goes hand in hand with task prompting. The qualitative data confirm these findings, showing a pattern in which the therapist first provides information about the module and subsequently prompts the patient to start the module. Unfortunately, the present findings do not allow us to detect whether informing, task prompting or a combination of the two is associated with decreased fatigue severity.

No significant associations were found between fatigue severity and the other seven therapist behaviours (emphatic utterances, probing self-reflection, psychoeducation, paraphrasing, task reinforcement, providing group context and alliance bolstering). It is possible that we have not found an association because our therapists already used these behaviours sufficiently. All behaviours occurred around once a week on average and this might be enough to be effective. In addition, the absence of an association with the outcome measure does not mean that the behaviour is not important. The behaviours may not be related to the reduction of fatigue, but could benefit the patient indirectly, for example by creating the willingness to continue to follow the therapy. Based on their expertise, we suggest therapists to apply the behaviours best suited for the situation and patient, while emphasizing informing and task prompting.

### 4.1 Strengths and limitations

The present study is the first to analyze online therapist behaviours in eMBCT for CCRF. By starting with a qualitative exploration of the data by two independent researchers, followed with a systematic coding of the data, we ensured a comprehensive set of therapist behaviours. Subsequently, we quantitatively examined the association between therapist behaviours and fatigue severity by means of linear regression analyses, while controlling for fatigue severity before starting the therapy. By combining qualitative and quantitative methods we provided a more complete understanding of therapists behaviours in eMBCT for CCRF.

The findings suggest that informing and task prompting are beneficial to outcome. Note that this is an association and we cannot be certain about its causal nature. To ensure causality a randomized design would be needed, where patients are exposed to different amounts of each therapist behaviour. Furthermore, it would be interesting to examine the most effective sequence of behaviours. When the patient first feels acknowledged and understood (e.g. by paraphrasing and emphatic utterances) the patient may then become more open to prompting and informing behaviours, increasing their effects. A second limitation of this study is the relatively small sample size (n = 31), resulting in a lack of power when using multivariate analysis. A larger sample size is needed in order to disentangle the role of informing and task prompting. A third limitation is that, while we coded the data as objectively as possible, it could be that the patient interpreted the therapist behaviours differently than we did. However, we tried to overcome this by identifying the therapist behaviours with two scientists reading the emails independently of each other. Lastly, we did not analyze what the patients wrote about their experiences and therefore did not have the context of the therapist's feedback.

### 4.2 Clinical implications

The present study suggests that when therapists put more informing and task prompting in their feedback in eMBCT for cancer patients, this is related to less CCRF at the end of the intervention. The implication for therapists is to make sufficient use of these behaviours.

## Declaration of competing interest

The authors declare that they have no known competing financial interests or personal relationships that could have appeared to influence the work reported in this paper.
